# An Unusual Case of an Immunocompetent Adult Diagnosed With Cytomegalovirus Colitis

**DOI:** 10.7759/cureus.58595

**Published:** 2024-04-19

**Authors:** Nilesh Jagdale, Mohith Prakash Kondapalli, Vutukuru Kalyan Kumar Reddy, Saimounika Adapa, Diksha Sabharwal

**Affiliations:** 1 General Medicine, Dr. D. Y. Patil Medical College, Hospital and Research Centre, Dr. D. Y. Patil Vidyapeeth, Pune, IND

**Keywords:** biopsy, colonoscopy, cytomegalovirus colitis, immunocompetent, immunocompromised

## Abstract

Cytomegalovirus (CMV) infections are typically considered opportunistic in individuals with immunosuppressive conditions such as human immunodeficiency virus/acquired immunodeficiency syndrome, underlying malignancies, organ or bone marrow transplantation, and those receiving chemotherapeutics or steroids. Nevertheless, there is a significant increase in reported instances of CMV infections globally, suggesting that even individuals with a healthy immune system might experience these infections. In such cases, the primary symptoms are typically related to the gastrointestinal system, such as ulcerative colitis, pseudopolyps, tumors, and ischemic and hemorrhagic enterocolitis. We present a unique instance of severe CMV colitis in a patient with a fully functioning immune system. The diagnosis of CMV colitis was determined through the histological analysis of biopsy tissues acquired during colonoscopy.

## Introduction

Cytomegalovirus (CMV) infections are common in immunosuppressive conditions, such as cancer, human immunodeficiency virus/acquired immunodeficiency syndrome, organ transplantation, and chemotherapy or steroids [1]. A growing number of cases worldwide show that CMV infections can also affect immunocompetent people and may present as ulcerative colitis, pseudopolyps, tumors, and ischemic and hemorrhagic enterocolitis [2].

The initial documentation of CMV colitis in an individual with a fully functioning immune system was recorded in 1992 [3]. Subsequently, certain pre-existing illnesses have been associated with a significant likelihood of CMV colitis in patients who do not appear to have weakened immune systems. These conditions include chronic renal insufficiency and hemodialysis, advanced age, co-infection with bacterial gastrointestinal infections, and food allergies [4,5].

CMV infections in individuals with a normal immune system can vary in severity, ranging from no symptoms to CMV-induced mononucleosis, pneumonitis, hepatitis, or colitis. However, silent cases are more common [6].

CMV impacts various organ systems, such as the gastrointestinal tract [7]. Gastrointestinal CMV infection is primarily observed in individuals with weakened immune systems who have luminal conditions, such as colitis or esophagitis. Nevertheless, there has been a growing prevalence of moderate-to-severe instances of colitis observed in both adults and children with a fully functioning immune system.

## Case presentation

A 22-year-old male patient came to our medical center with the chief complaints of fever, loose stools, abdominal pain, and hematochezia for the last 15 days. There was no history of melena, vomiting, edema, rashes, or petechiae. The patient had no other history of comorbidities. The patient’s appetite had reduced since the onset of the above symptoms. The patient had a history of eating outside food 15 days ago and has lost 3-4 kg of weight in the last 15 days.

Upon examination, blood pressure was 110/70 mmHg, pulse rate was 112 beats/minute, oxygen saturation was 98%, and respiratory rate was 18 breaths/minute. On palpation, epigastric tenderness was present, and there was no organomegaly. On auscultation, reduced bowel sounds were heard. Routine blood investigations are presented in Table 1, and routine stool examination findings are presented in Table 2.

**Table 1:** Routine blood investigations. SGOT: serum glutamic-oxaloacetic transaminase; SGPT: serum glutamic pyruvic transaminase

Investigation	Normal range	Day 1	Day 5	Day 10	Day 15	Day 20	Day 25
Hemoglobin	14–17 g/dL	11	8.80	9.10	8.40	9.40	10.70
Total leukocyte count	4,000–11,000 mm^3^/µL	18,900	13,400	12,800	9,000	8,800	7,500
Platelets	150,000–410,000/µL	345,000	289,000	300,000	270,000	240,000	375,000
Serum bilirubin	0.2–1.2 mg/dL	1.2	2.6	2.5	2.0	1.5	1.1
SGOT	8–48 IU/L	45	70	62	23	20	25
SGPT	7–55 IU/L	67	200	135	124	67	42
Serum urea	17–49 mg/dL	35	78	56	45	40	30
Serum creatinine	0.6–1.35 mg/dL	0.59	1.45	1.23	1.20	1.10	0.78
Serum sodium	135–145 mmol/L	130	124	127	130	131	136
Serum potassium	3.5–5.1 mmol/L	3.70	3.25	2.90	3.45	3.60	3.78

**Table 2:** Stool routine examination. Hpf: high-power field

Stool examination	Day 1	Day 5	Day 10	Day 15	Day 20	Day 25
Colour	Reddish brown	Reddish brown	Reddish brown	Brown	Yellow	Yellow
Mucus	Present	Present	Present	Present	Absent	Absent
Occult blood	Present	Present	Present	Absent	Absent	Absent
Pus cells	Plenty	Plenty	20–40/hpf	10–15/hpf	2–3/hpf	2–3/hpf
Red blood cells	Plenty	Plenty	30–50/hpf	12–15/hpf	2–4/hpf	Absent
Cysts	Absent	Absent	Absent	Absent	Absent	Absent
Ova	Absent	Absent	Absent	Absent	Absent	Absent
Larvae	Absent	Absent	Absent	Absent	Absent	Absent
Parasites	Absent	Absent	Absent	Absent	Absent	Absent

**Figure 1:**
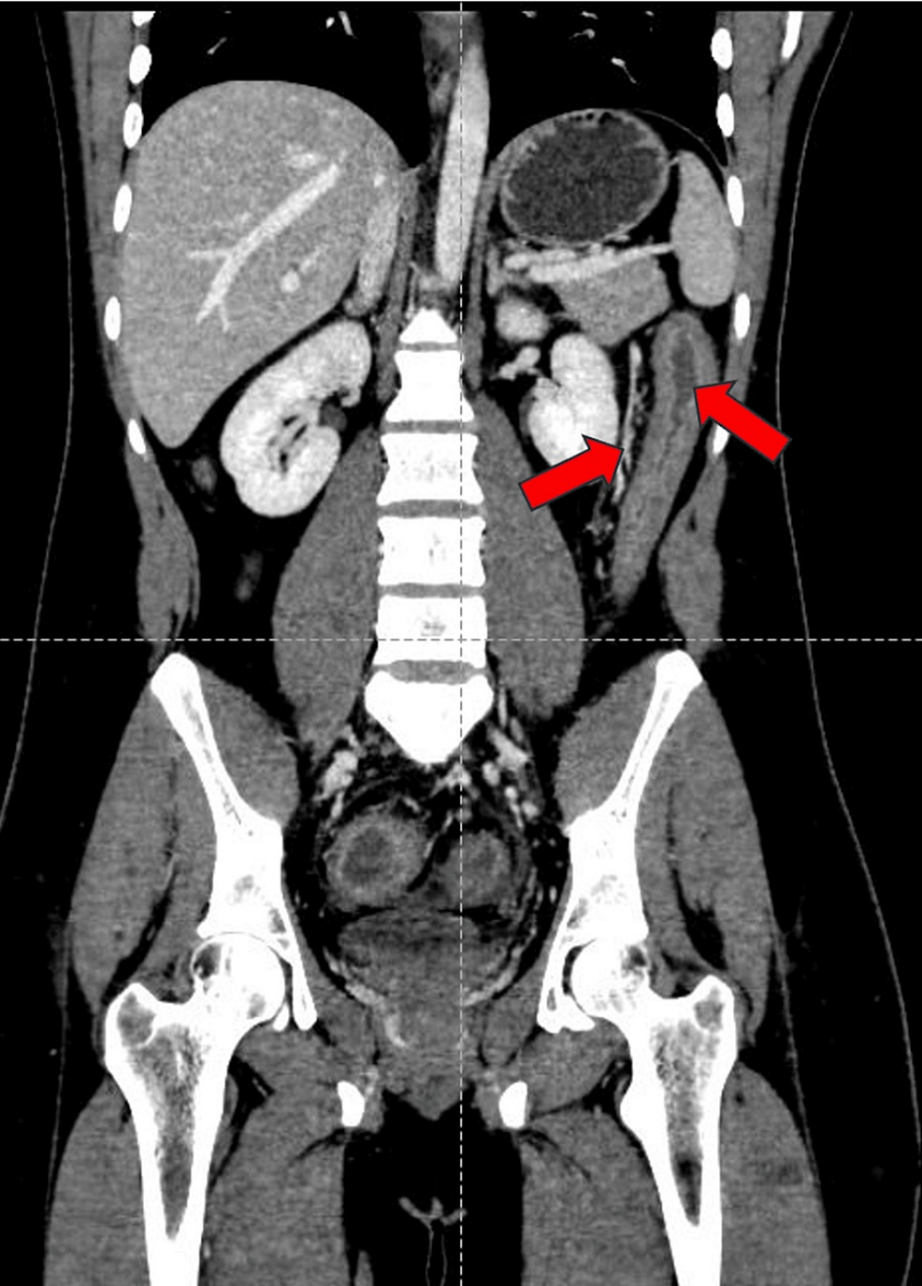
CECT-A/P coronal section: diffuse wall thickening involving the descending colon with serosal hyperemia (red arrows). CECT-A/P: contrast-enhanced computed tomography of the abdomen and pelvis

**Figure 2:**
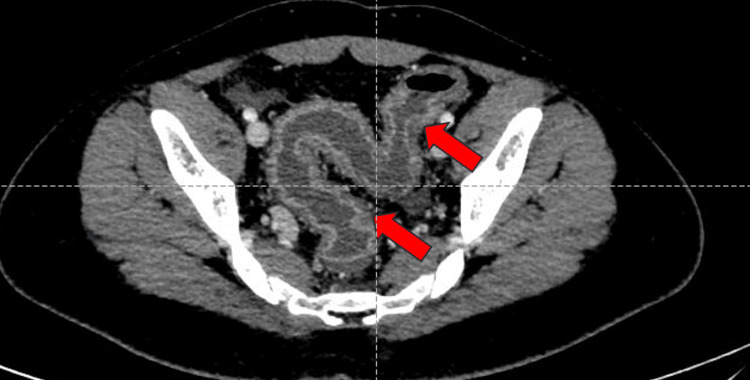
CECT-A/P axial section: diffuse wall thickening of the sigmoid colon and rectum with surrounding fat stranding and vessel engorgement (red arrows). CECT-A/P: contrast-enhanced computed tomography of the abdomen and pelvis

Contrast-enhanced computed tomography of the abdomen and pelvis (CECT-A/P) revealed diffused wall thickening of the entire large bowel, with the maximum wall thickness measuring approximately 9 mm. Colonic submucosal edema and areas of fat deposition with mucosal and serosal hyperemia (Figure 1) were noted, giving a target appearance predominantly involving hepatic flexure, transverse colon, descending colon, and sigmoid colon. Surrounding fat stranding and vessel engorgement were seen (Figure 2). Loss of normal haustrations was seen in the descending colon. The bowel loops appeared normal in caliber. Given these findings, differential diagnoses of inflammatory colitis or infective colitis were made.

**Figure 3:**
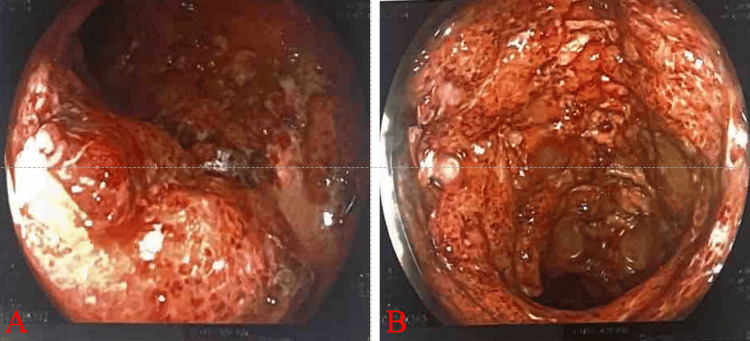
Colonoscopy: the visualized mucosa showing severe erythema, loss of vascularity, multiple superficial to deep ulcerations, nodularity, and friability at the descending colon (A) and rectum (B).

Colonoscopy revealed that the visualized mucosa had severe erythema, loss of vascularity, multiple superficial to deep ulcerations, nodularity, and friability (Figures 3A, 3B).

**Figure 4:**
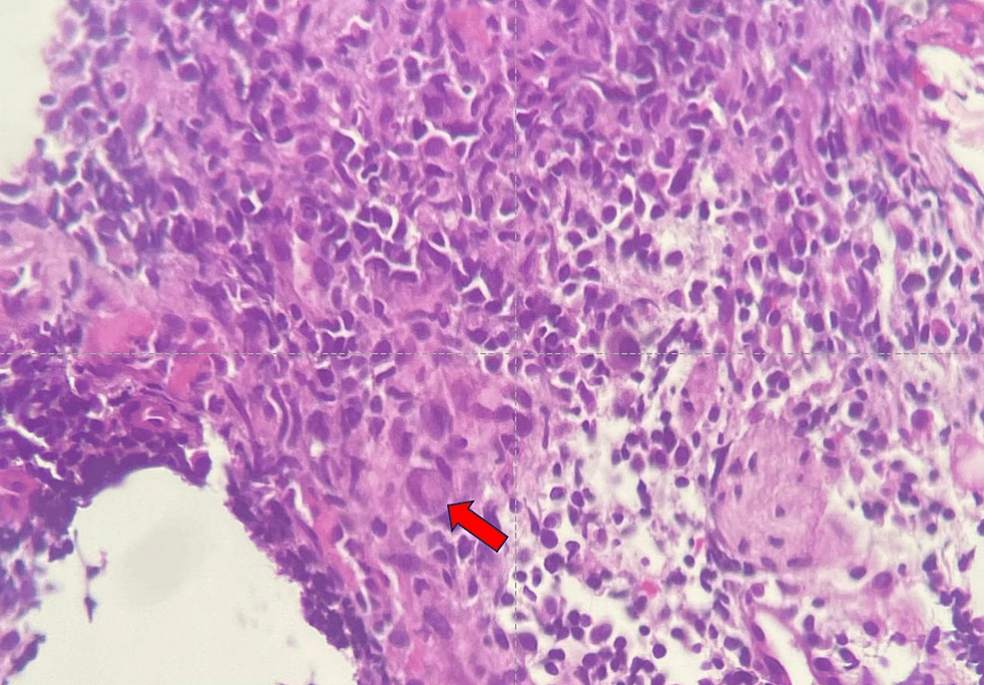
Colonic biopsy section showing multiple fragments of the colonic mucosa with a disrupted architecture: a large ovoid or pleomorphic nucleus with basophilic intranuclear inclusions (Cowdry bodies) surrounded by a clear halo suggestive of CMV colitis at 40× magnification (H&E stain). CMV: cytomegalovirus; H&E: hematoxylin and eosin

A biopsy was taken from the colon and sent for histopathological examination, which revealed multiple fragments of the colonic mucosa with disrupted architecture. A large ovoid or pleomorphic nucleus with basophilic intranuclear inclusions (Cowdry bodies) surrounded by a clear halo suggestive of CMV colitis at 40× magnification, hematoxylin and eosin stain (Figure 4), and a positive CMV polymerase chain reaction. The stool BioFire film array gastrointestinal panel was negative (Table 3).

**Table 3:** Stool BioFire film array gastrointestinal panel.

	Result
*Campylobacter*	Not detected
*Clostridium difficile* (A/B)	Not detected
*Salmonella*	Not detected
*Vibrio cholera*	Not detected
Enteroaggregative *Escherichia coli*	Not detected
Enterotoxigenic *Escherichia coli*	Not detected
Shiga-like toxin *Escherichia coli*	Not detected
Astrovirus	Not detected
Norovirus	Not detected
Adenovirus (40/41)	Not detected

Based on these reports, a diagnosis of CMV colitis was made. Intravenous (IV) ganciclovir 5 mg/kg was given for 21 days. The patient improved and was discharged on the 25th day, and the follow-up was uneventful.

## Discussion

CMV is a member of the herpes virus family that has a double-stranded DNA structure. The incidence of CMV infections in the overall adult population ranges from 40% to 100% [8]. CMV infections are prevalent globally as a result of their release in bodily fluids and spread through intimate contact [9]. Primary infection in typical hosts is typically asymptomatic; however, it can occasionally lead to a mononucleosis-like syndrome. This condition is characterized by symptoms such as fever, muscle pain, swelling of the lymph nodes in the neck, and increased levels of liver enzymes [9].

CMV colitis manifests with symptoms such as fever, weight loss, lack of appetite, general discomfort, and pain in the abdomen. In addition, watery diarrhea and hematochezia are commonly observed symptoms. Mucosal bleeding and perforation are severe consequences of CMV colitis that can be life-threatening [10].

CMV initiates a primary infection, which is then followed by a latent phase. Recurrent illness may manifest when the virus becomes active again as a result of compromised immunity caused by factors such as advanced age or immunosuppressive medications. CMV is prevalent, with a seroprevalence immunoglobulin G positivity ranging from 40% to 100% in adults, and this prevalence tends to increase as individuals age [11].

Of the 44 individuals identified with CMV colitis in a recent study, only 10 did not have any other medical issues, which is a significant finding. Nevertheless, the remaining 34 patients exhibited several comorbid conditions that hindered the function of the host defense system, such as pregnancy, renal illness, diabetes, and malignancy [12].

CMV colitis can be attributed to two mechanisms, namely, primary and secondary. CMV is the primary factor responsible for the proliferation and inflammation of endothelial cells, rendering them susceptible to vasculitis, ulcers, and ultimately ischemic colitis [13]. The secondary mechanism arises from pre-existing disorders, such as inflammatory bowel disease (IBD) or ischemic colitis, which can result in damage to the mucosal lining and subsequent local immune suppression. These circumstances may enable the coexistence of CMV infection with IBD or ischemic colitis. Our patient exhibited acute hemorrhagic colitis before the onset of the CMV infection, despite the initial CECT-A/P scan indicating minor symptoms. However, later biopsy specimens revealed the presence of CMV inclusion bodies. Early diagnosis and prompt treatment of CMV colitis generally result in a favorable prognosis.

Patel et al. found that CMV colitis commonly affects the sigmoid colon and rectum in immunocompetent patients. Endoscopic examinations frequently reveal the presence of ulceration [14]. At times, the presence of pseudo-membrane and pseudo-polyps might be observed, which can complicate the accurate diagnosis of CMV colitis as pseudo-membranous colitis.

Ganciclovir is currently the preferred antiviral medication for treating CMV infection in individuals with a healthy immune system. Even valganciclovir and foscarnet have been shown to significantly enhance the prognosis of patients with CMV colitis [15-17]. Nevertheless, ganciclovir has the potential to cause severe negative effects, such as myelosuppression, central nervous system problems, hepatotoxicity, and nephrotoxicity [9].

Although antiviral therapy might produce significant adverse effects, it is advisable for immunocompetent patients due to the unfavorable consequences of CMV colitis in the absence of such treatment [12].

## Conclusions

Patients with CMV colitis have nonspecific symptoms such as diarrhea, pain in the abdomen, fever, bleeding per rectum, and weight loss. Hematochezia and diarrhea are among the most common symptoms in such individuals, as observed in our case. As a result, a high index of suspicion is required. Laboratory studies are critical in identifying CMV colitis. CMV colitis can be diagnosed using biopsies, colonoscopy, or a CT scan. Prompt identification and intervention are crucial to enhancing the prognosis of patients. Ultimately, it is important to acknowledge CMV colitis as a potential medical condition, not just in individuals with weakened immune systems but also in individuals with normal immune function, particularly after ruling out more prevalent causes of severe diarrhea.
